# Re-establishment of Carabus (Cathoplius) aliai Escalera, 1944 as a separate valid species (Coleoptera, Carabidae)

**DOI:** 10.3897/zookeys.496.9428

**Published:** 2015-04-16

**Authors:** Claudio Ghittino, Enrico Busato, Achille Casale

**Affiliations:** 1 Istituto Zooprofilattico Sperimentale dell’Umbria e delle Marche (IZSUM), Via Dalla Chiesa 78, 05100 Terni, Italy; 2 Dipartimento di Scienze Agrarie, Forestali e Alimentari (DISAFA), Università di Torino, Largo Braccini 2, 10095 Grugliasco (TO), Italy; 3 c/o Dipartimento di Scienze della Natura e del Territorio (DIPNET), sezione Zoologia, Università di Sassari, Via Muroni 25, 07100 Sassari, Italy

**Keywords:** *Carabus* ground beetles, Saharan desert endemism, Atlantic element, life cycle, hybridization

## Abstract

Carabus (Cathoplius) aliai was described as a separate species by Escalera in 1944 but since the 1950–60s it has been considered as a subspecies of Carabus (Cathoplius) stenocephalus Lucas, 1866. This downgrading was adopted after examining only a few specimens, due to their rarity in collections. In recent years, an important population of this taxon was rediscovered in the Tan-Tan area in southern Morocco. By combining field observations with laboratory breeding experiments including hybridization trials, and through the morphological examination of a representative number of individuals, it is confirmed that *Carabus aliai* is indeed a valid species. Despite close geographic distribution, the morphological and biological characteristics of *Carabus aliai* and *Carabus stenocephalus ifniensis* Zarco, 1941, its northern substitutive taxon, are very different. *Carabus aliai* adults are characterized by a smaller size, a slender silhouette, a more brilliant aspect, a narrower pronotum, a coarser elytral sculpture, longer legs, and a wider and a little more curved apex of the median lobe of the aedeagus. *Carabus aliai* larvae are also characterized by a much smaller size and the *Carabus aliai* pupa has a narrower thoracic area and a different chaetotaxy compared to that of *Carabus stenocephalus ifniensis*. Contrary to this, *Carabus aliai* has a life cycle belonging to the annual univoltine winter semelparous type. Moreover, the duration of its development cycle is shorter. *Carabus aliai* is a sabulicolous steppe-wandering species with an intensive running activity, while *Carabus stenocephalus ifniensis* is a more sedentary taxon. Crossbreeding experiments showed a marked reproductive isolation between *Carabus aliai* and *Carabus stenocephalus ifniensis*. When F1 hybrids were crossed with one another, a very high mortality rate during embryonic, larval and pupal development was evident and no vital F2 neo-adults were obtained. Morphological and biological differences, together with the reproductive failure in *Carabus aliai* × *Carabus stenocephalus ifniensis* hybrids, clearly indicate that *Carabus aliai* is a separate *Cathoplius* species that is distributed in an area south of the Anti-Atlas chain, from Plage Blanche (Guelmim) to Lemsid and Bou Kra (south of Laâyoune). *Carabus aliai* is therefore both a Saharan desert endemic and an Atlantic resident. Moreover, it is the southernmost *Carabus* species of the western Palaearctic region.

## Introduction

The subgenus *Cathoplius* C.G. Thomson, 1875 within the Genus *Carabus* Linnaeus, 1758 forms a very homogenous and peculiar lineage of strictly snail-predating ground beetles adapted to live in arid habitats with scarce xerophilous vegetation along the Moroccan Atlantic coast (Busato et al. 2014). This subgenus has been postulated as the only representative of Cathopliogenici, a possible sister group of the “Neocarabi” of Bengtsson (Deuve et al. 2012).

Carabus (Cathoplius) aliai is a desert-dwelling taxon that was first described by Manuel Martinez de la Escalera in August 1944. This description was based on the examination of only one specimen, a female found by the Spanish geologist Manuel Alía in April 1943 in El Khaloua which is 15 km north of present Abteh, in former Cape Juby strip, southern territories of the Spanish protectorate in Morocco (Escalera 1944). At approximately the same time (September 1944), it was also described by Francisco Español and named as Carabus (Cathoplius) mateui. This description was based on three specimens, a female found by Joaquin Mateu in February 1944 in Agti Baba Ali in the Sabkhat Tislatine area, 50 km southwest of present Laâyoune, in former Saguia el Hamra, Spanish Sahara, and a male and a female found in May 1944 in Tan-Tan, in former Cape Juby strip, in the southern territories of the Spanish protectorate in Morocco (Español 1944).

Dr Joaquin Mateu, to whom this contribution is dedicated, really discovered and first studied *Carabus aliai*. In November 1944, he collected a further seven specimens (five males and two females) south of Laâyoune, in an area comprised between Izik plateau, Lemsid (Ougnit) and Metmarfag (Asreifa) to the west and Bou Kra to the east. This area is characterized by the presence of “graras”, land depressions where water cumulates during the raining season, allowing a certain vegetation to grow and consequently snails to dwell in. It was here that *Carabus aliai* specimens were observed during early morning or late afternoon while running, mating or eating snails of the family Helicidae. In addition to the biogeographic and biological notes, in his paper on the carabid beetles of Spanish Sahara, Mateu (1947) established *Carabus mateui* as a junior synonym of *Carabus aliai*.

Some years later, French entomologists dealing with the Moroccan carabid fauna reconsidered *Carabus aliai* as a subspecies of Carabus (Cathoplius) stenocephalus Lucas, 1866 (Antoine 1955; Kocher 1963). This downgrading was adopted after examining one of the specimens collected by Mateu in 1944, plus another doubtful specimen found by André Reymond in 1954 at Aoreora, Plage Blanche (75 km west of Guelmim / 30 km north of the Oued Drâa outlet, in the southern part of the former French protectorate in Morocco). As far as we know, few other specimens of *Carabus aliai* have been found until December 2003, when several individuals of this taxon were sampled by Jaroslav Kaláb in the vicinity of the city of Tan-Tan, Tan-Tan province, southern Morocco.

Modern catalogues and checklists of the Genus *Carabus* either consider *Carabus aliai* as a “strong” subspecies of Carabus (Cathoplius) stenocephalus (Deuve 1994; 2004; 2012; 2014), together with the “weak” subspecies *escalerae* Csiki, 1927, *susicus* Antoine, 1941 and *ifniensis* Zarco, 1941, or as a subspecies of Carabus (Cathoplius) asperatus (Dejean, 1826) in the broad sense (Březina 1994; 1999; Bousquet et al. 2003; Lorenz 2005). In this study, we collected data from field observations and laboratory breeding experiments, including hybridization trials with the northern substitutive taxon *Carabus stenocephalus ifniensis*. By combining this information with morphological examinations of a representative number of individuals, both pre–imaginal stages and adults, it was concluded that *Carabus aliai* is indeed a separate species, as considered in the original descriptions (Escalera 1944; Español 1944) and as hypothesized in our previous contribution (Busato et al. 2014).

## Methods

Field observations on Carabus (Cathoplius) aliai Escalera, 1944 are the results of nine surveys (March–April 1992; December 1999; January 2000; January, February–March & April 2006; November 2008; January & December 2010) carried out in the Guelmim, Tan-Tan and Laâyoune areas, southern Morocco. From December 14–19, 2010, one of the authors (C. G.) sampled 11 adult specimens (7 males and 4 females) and 10 larvae (1 first instar and 9 second instars) of this taxon on the Tan-Tan northern plateau (Hameidia Tellia: 2 km west of Tan-Tan city, 90–110 m a.s.l., 28°27'13"N; 11°07'53"W, and 7 km west of Tan-Tan city, 190 m a.s.l., 28°26'21"N; 11°10'30"W, in Ben Khlil rural commune, Tan-Tan province). In addition, observations on the behavior of specimens in their natural environment were recorded.

Life cycle investigations included the maintenance and breeding of *Carabus aliai* adults and larvae in the laboratories of the State Veterinary Institute (IZSUM) in Terni, Italy. Breeding activities started on 21 December 2010 and ended on 30 April 2013. The breeding methodology used was basically that reported by Malausa (1977). Insects were exposed to varying temperatures (from 15/19 °C in December to 17/21 °C in April, night/day) and to a gradually increasing photoperiod (from 9 to 14 hours of daylight).

*Carabus aliai* mating pairs were maintained in a 50 × 30 × 30 cm (length × width × height) transparent terrarium with a perforated top for aeration. A 15 cm substrate, composed of a mixture of argillaceous soil (40%) and sand (60%) was placed on the bottom of each terrarium. One third of the substrate surface was covered with a layer of moss. The substrate was kept moist by periodic spraying with water. Adults were *ad libitum* fed with live *Theba pisana pisana* (O.F. Müller, 1774) snails. In order to compare their life cycles, Carabus (Cathoplius) stenocephalus ifniensis adults (previously collected on the beach of Sidi Ifni city, 10 m a.s.l., 29°23'52"N; 10°10'40"W, Sidi Ifni province, central Morocco) were bred in parallel with *Carabus aliai*. Two mating pairs of both taxa were introduced in the terrariums on 21 December 2010 and two others on 1 December 2011 as a control group.

Larvae were reared individually in cylindrical opaque plastic containers (10 cm in diameter, 13 cm height) filled with 9 cm of the same substrate and kept at the same temperature as adults. Larvae were fed with live *Theba pisana pisana* snails of the appropriate size (shell width 10–20 mm). In each container, a piece of bark [5 × 3 × 1(h) cm] was placed on top of the substrate as a shelter for the larva. Neo-adults were kept in mini-terrariums [20 × 12 × 15(h) cm] with a 5 cm substrate and a layer of moss, and *ad libitum* fed with live *Theba pisana pisana* snails until their complete sclerification.

Following pure breeding, hybridization experiments between virgin *Carabus aliai* and *Carabus stenocephalus ifniensis* were carried out. Four mating pairs of the combination *Carabus aliai* ♂ × *Carabus stenocephalus ifniensis* ♀ (AIF1) and four of the combination *Carabus stenocephalus ifniensis* ♂ × *Carabus aliai* ♀ (AIF2) were bred at the same temperatures and at the same photoperiod previously reported, starting from December 1, 2011. Due to the different size between *Carabus aliai* (generally smaller) and *Carabus stenocephalus ifniensis* (generally larger), small females of *Carabus stenocephalus ifniensis* and large males of *Carabus aliai* were used to obtain AIF1 hybrid, while large females of *Carabus aliai* and small males of *Carabus stenocephalus ifniensis* were used to obtain AIF2 hybrid. Once obtained, AIF1 and AIF2 hybrids were then crossed with one another to investigate their fecundity. The F1 crossbreeding experiments were done in quadruplicate starting on December 3, 2012.

Representative specimens of *Carabus aliai* and *Carabus stenocephalus ifniensis* used for comparative morphological studies are conserved dry or under alcohol in authors’ collections.

## Results

**Figures 1–6.**
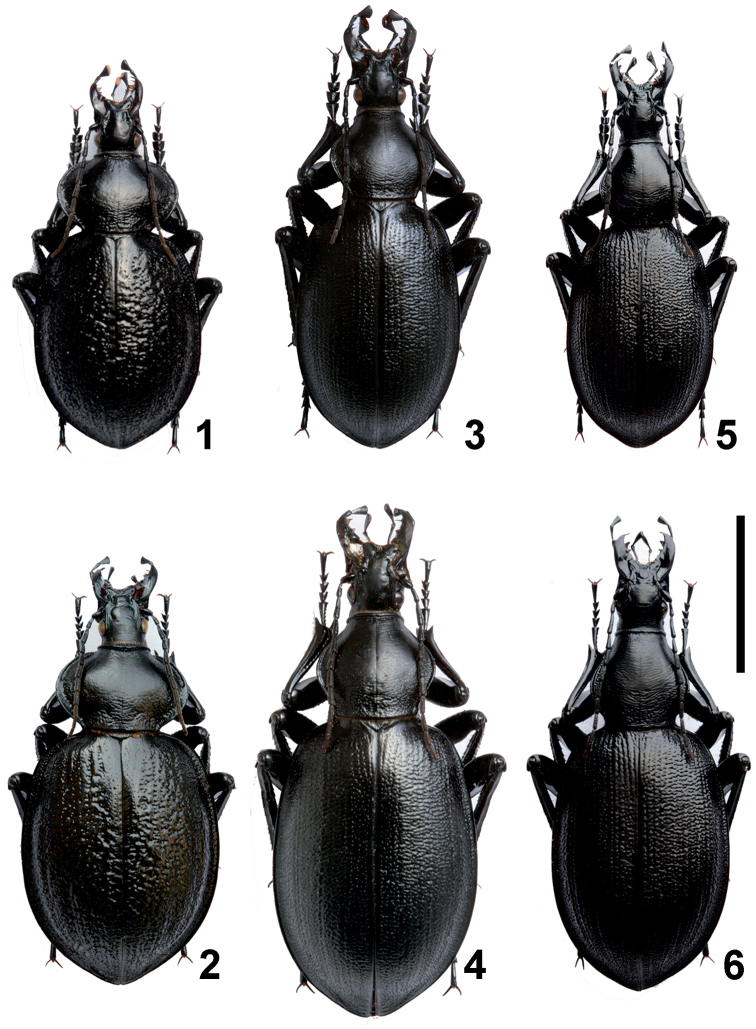
*Cathoplius* species adults. Habitus of *Carabus asperatus* (Dejean, 1826) from Oualidia (**1** male **2** female) *Carabus stenocephalus ifniensis* Zarco, 1941 from Sidi Ifni (**3** male **4** female) and *Carabus aliai* Escalera, 1944 from Tan-Tan (**5** male **6** female). Scale bar: 1 cm.

***Morphology of adults.*** Morphological differences observed among adults of the various *Cathoplius* taxa were in agreement with those reported by Antoine (1955) (Figures 1–6). The size of Carabus (Cathoplius) aliai Escalera, 1944 and Carabus (Cathoplius) stenocephalus ifniensis Zarco, 1941 adults was found to be a peculiar feature: *Carabus aliai* specimens were in general smaller (shorter and narrower), while specimens of its northern substitutive *Carabus stenocephalus ifniensis* were larger (longer and wider). The total length from apex of mandibles to apex of elytra (L) was 26.5–33.0 mm in *Carabus aliai* (males 26.5–28.5 mm, females 27.0–33.0 mm) and 28.0–36.0 mm in *Carabus stenocephalus ifniensis* (males 28.0–30.0, females 30.0–36.0 mm). The general aspect was also found to be very peculiar: *Carabus aliai* specimens were bright black in color on their dorsal side, while specimens of *Carabus stenocephalus ifniensis* were dull black.

The head was slender in *Carabus aliai* and broad in *Carabus stenocephalus ifniensis*. The pronotum was narrow and elongated, longer than wide, with a very reduced lateral furrow in *Carabus aliai*, while it was transverse, wider than long, with a developed lateral furrow in *Carabus stenocephalus ifniensis*. On average, the ratio between length of pronotum and maximum width of pronotum (PL/PW) was 6.0/5.5 mm = 1.09 in *Carabus aliai* and 5.5/7.0 mm = 0.79 in *Carabus stenocephalus ifniensis*. In both taxa the prosternal apophysis was elongated, but while its apex was subquadrate in *Carabus stenocephalus ifniensis* it was rounded in *Carabus aliai*. The elytra were parallel-sided and flattened in *Carabus aliai*, ovoid and convex in *Carabus stenocephalus ifniensis*. On average, the ratio between length of elytra and maximum width of elytra (EL/EW) was 17.5/11.5 mm = 1.52 in *Carabus aliai* and 19.5/13.0 mm = 1.50 in *Carabus stenocephalus ifniensis*. In females of both taxa, the apex of the elytra was more acuminate than the one of males. The elytral sculpture was composed by deep striae with coarse punctuation in *Carabus aliai* and by slightly marked striae with fine punctuation in *Carabus stenocephalus ifniensis*. Legs were proportionally longer in *Carabus aliai* than in *Carabus stenocephalus ifniensis*.

**Figures 7–14.**
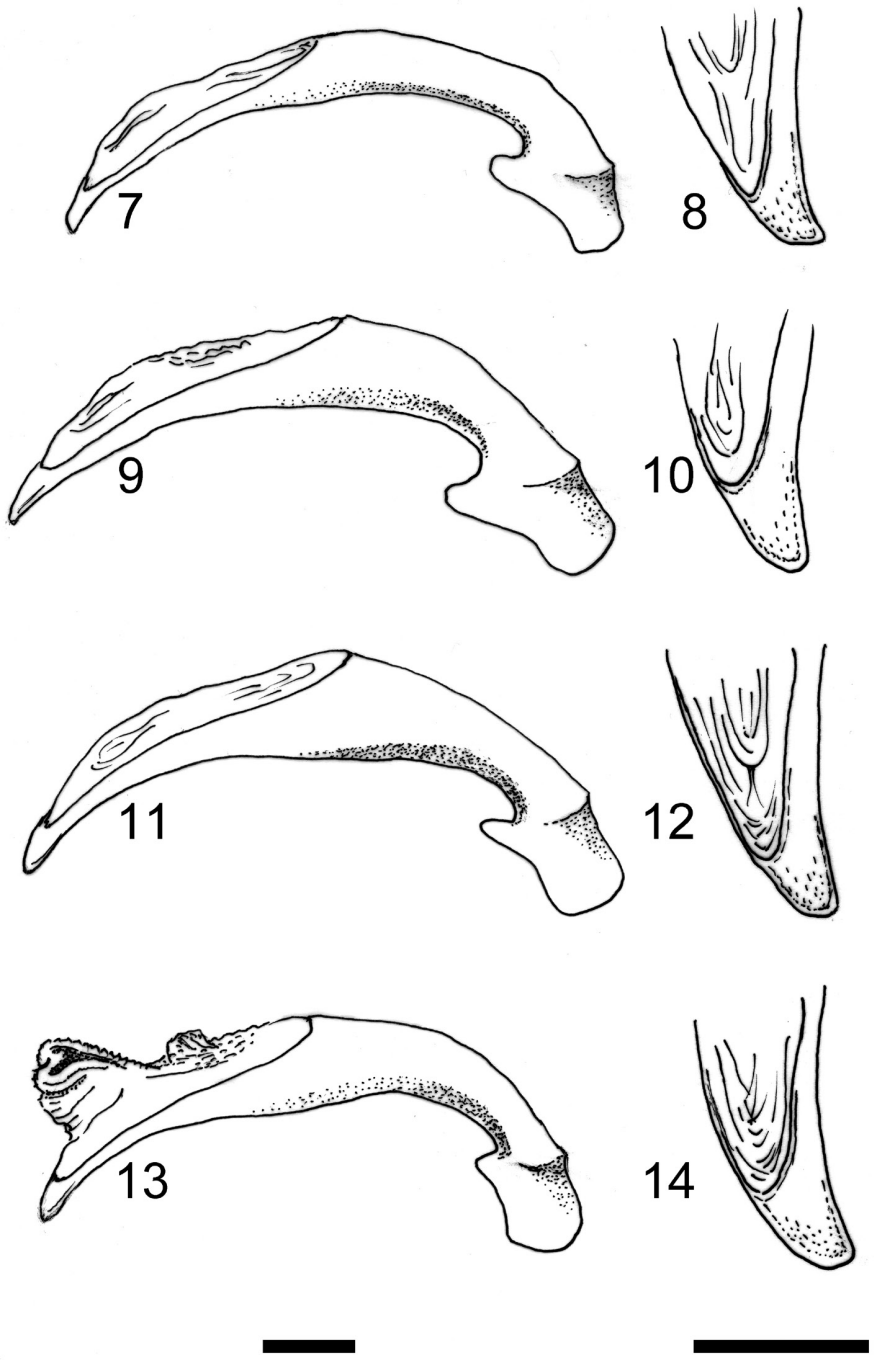
*Cathoplius* species aedeagi. Right lateral aspect of the median lobe (left) and details of its apex (right) in *Carabus asperatus* from Oualidia (**7–8**) *Carabus stenocephalus susicus* from Aglou (**9–10**) *Carabus stenocephalus ifniensis* from Sidi Ifni (**11–12**) and *Carabus aliai* from Tan-Tan (**13–14**). Scale bars: 1 mm.

Both male and female genitalia were found to be very homogeneous within representatives of subgenus *Cathoplius*. The median lobe of the aedeagus in all taxa was similar either in size or in shape (Figures 7–14). In *Carabus asperatus* (Dejean, 1826) the median lobe was slightly smaller, more elongate and its apex was more acutely shortened, while in *Carabus aliai* Escalera, 1944 the apex was wider and a little more curved than in *Carabus stenocephalus susicus* Antoine, 1941 and *Carabus stenocephalus ifniensis* Zarco, 1941. Parameres were similar.

**Figures 15–19.**
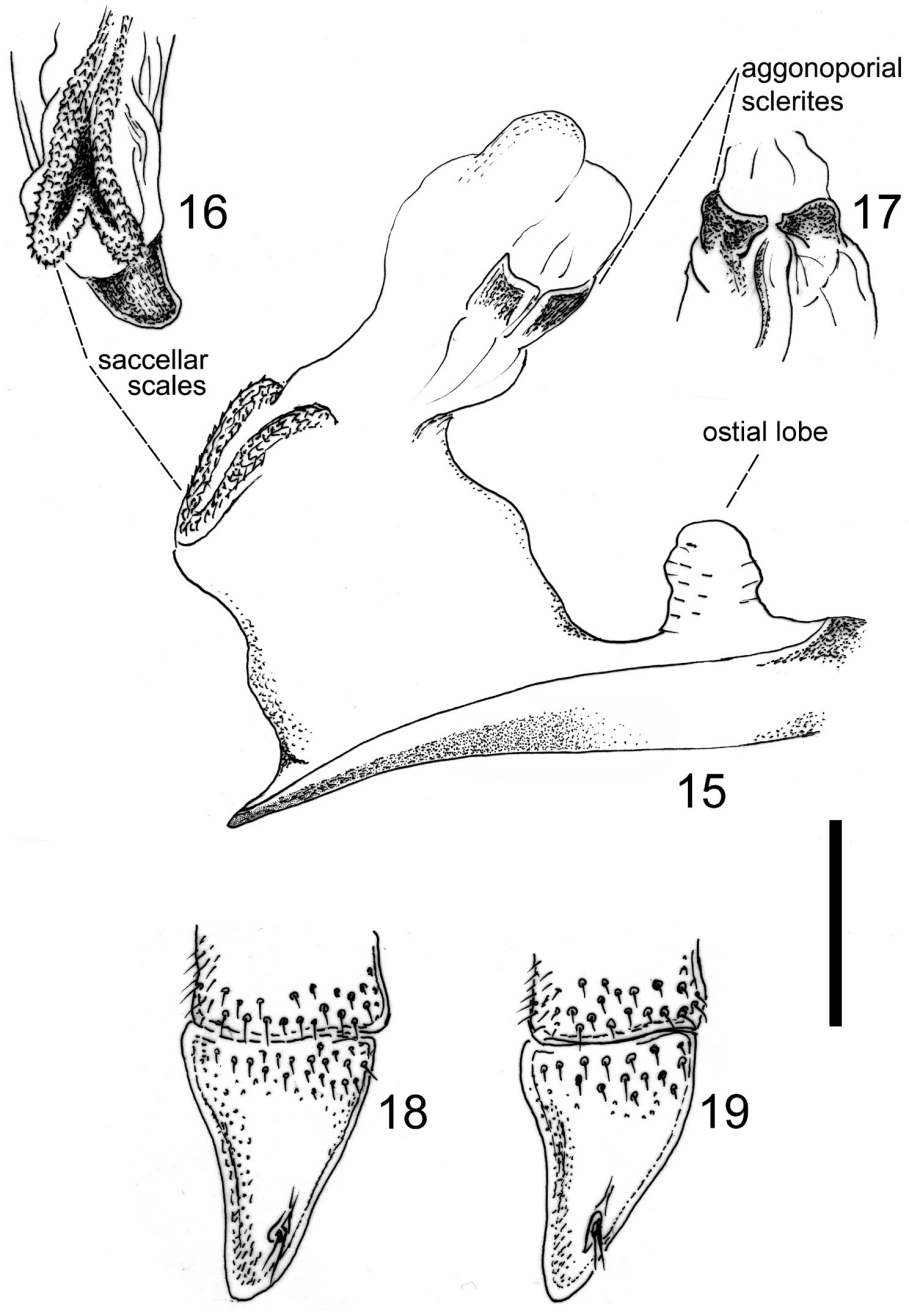
*Cathoplius* species male and female genitalia. Endophallus of *Carabus asperatus* from Oualidia, with emphasis on the aggonoporius, saccellus and ostial lobe (**15**). Details of the saccellar (**16**) and aggonoporial (**17**) areas in *Carabus aliai* from Tan-Tan. Gonocoxite 2 of the ovipositor in *Carabus asperatus* from Oualidia (**18**) and in *Carabus aliai* from Tan-Tan (**19**). Scale bar: 1 mm.

The endophallus in *Cathoplius* species showed a peculiar morphological feature (Figures 15–17). In the distal aggonoporial area, two small sclerites, slightly different in *Carabus aliai* and in *Carabus stenocephalus* from those of *Carabus asperatus*, were present. In the saccellar area, a tumid, v-shaped body covered with dense, prominent scales and spines, was evident. The ostial lobe was well developed. The female inner reproductive tract of the various *Cathoplius* taxa was found to be identical, being fully membranous with no sclerified structures. External genitalia, in the specific gonocoxite 2 of the ovipositor, were also very similar in all species (Figures 18–19).

**Figures 20–23.**
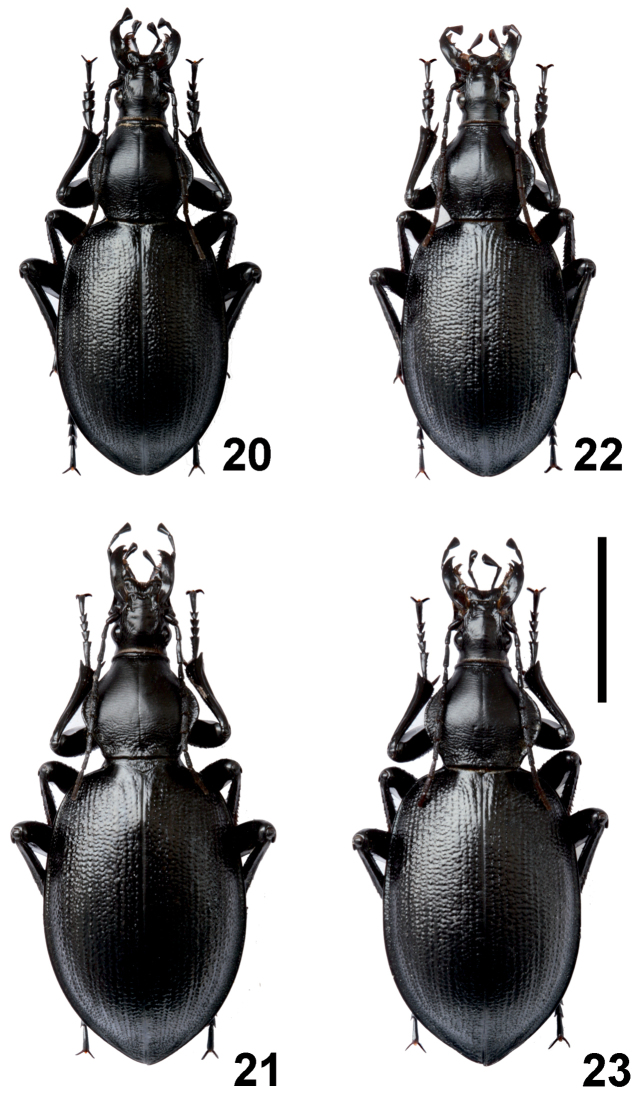
Carabus (Cathoplius) aliai Escalera, 1944 × *Carabus stenocephalus ifniensis* Zarco, 1941 hybrids. Habitus of hybrid AIF1 (**20** male **21** female) and hybrid AIF2 (**22** male **23** female) from our laboratories. Scale bar: 1 cm.

*Carabus aliai* × *Carabus stenocephalus ifniensis* hybrids showed intermediate features when compared to parental species. Hybrids were morphologically more similar to the parent female than to the male: hybrid AIF1 (♂ *Carabus aliai* × ♀ *Carabus stenocephalus ifniensis*) (L 28.0–36.0 mm) was a little more similar to *Carabus stenocephalus ifniensis* than hybrid AIF2 (♂ *Carabus stenocephalus ifniensis* × ♀ *Carabus aliai*) (L 27.0–34.0 mm) which in turn was more similar to *Carabus aliai* (Figures 20–23).

***Morphology of pre-imaginal stages.*** Morphological differences observed among the pre–imaginal stages of the various *Cathoplius* taxa were corresponding to those reported by Busato et al. (2014). Carabus (Cathoplius) aliai Escalera, 1944 larvae were found to be very small, being much shorter and more slender than the ones of Carabus (Cathoplius) stenocephalus ifniensis Zarco, 1941. Their total length varied according to the development period and to the quantity of food ingested. Minimum and maximum length and width of the three larval instars of *Carabus aliai* and *Carabus stenocephalus ifniensis* are reported in Table 1.

**Figure 24.**
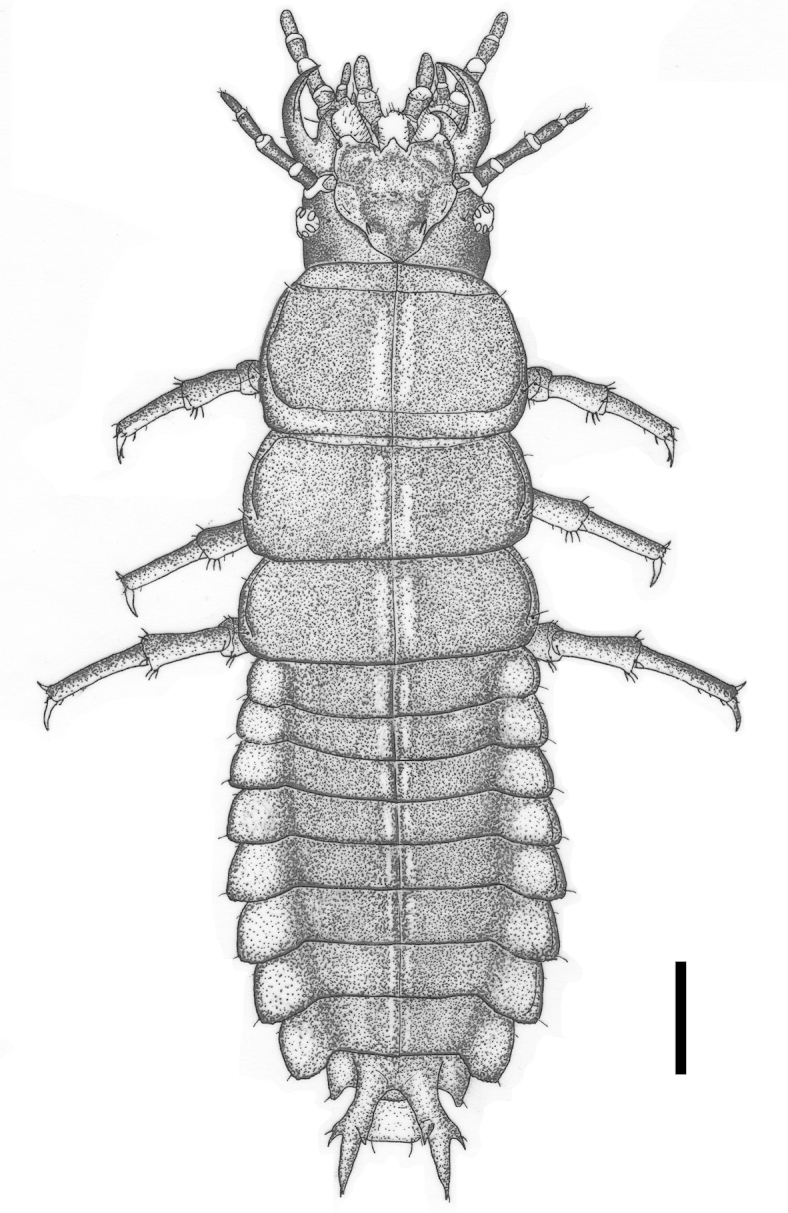
Carabus (Cathoplius) aliai Escalera, 1944 first instar larva: dorsal aspect. Scale bar: 1 mm.

The *Carabus aliai* newborn larvae (Figure 24) measured 9.5 × 2.0 mm (length × width), versus 11.0 × 2.5 mm in *Carabus stenocephalus ifniensis*. Before burying, the *Carabus aliai* third instar larvae measured 26.0–27.0 × 6.5 mm (males) or 29.0–30.0 × 6.5 mm (females), versus 27.0–29.0 × 7.0 mm (males) or 32.0–34.0 × 7.0 mm (females) in *Carabus stenocephalus ifniensis*. In both taxa, the length/width ratios of the *frontoclypeolabrum* of the three larval instars were similar. For the size difference, appendages (antennae, mouthparts and legs) looked shorter in *Carabus aliai* than in *Carabus stenocephalus ifniensis* but length ratios between segments were similar.

**Table 1.** Size of Carabus (Cathoplius) aliai Escalera, 1944 and Carabus (Cathoplius) stenocephalus ifniensis Zarco, 1941 pre–imaginal stages.

Pre–imaginal stages	*Carabus aliai* (length × width, mm)	*Carabus Carabus ifniensis* (length × width, mm)
1^st^ instar larva:		
- newborn	9.5–10.0 × 2.0	11.0–11.5 × 2.5
- before ecdysis	15.0–16.5 × 2.5	16.5–18.0 × 3.0
2^nd^ instar larva:		
- after ecdysis	16.0–16.5 × 3.5	17.5–18.0 × 4.0
- before ecdysis	20.0–21.5 × 4.5	23.0–24.5 × 5.0
3^rd^ instar larva:		
- after ecdysis	21.0–23.0 × 6.0	24.0–26.0 × 6.0
- before burying	26.0–30.0 × 6.5	28.0–34.0 × 7.0
- pre–pupa	23.0–26.0 × 6.5	25.0–29.0 × 7.0
Pupa	20.0–22.0 × 9.0	22.0–25.0 × 9.5

**Figures 25–27.**
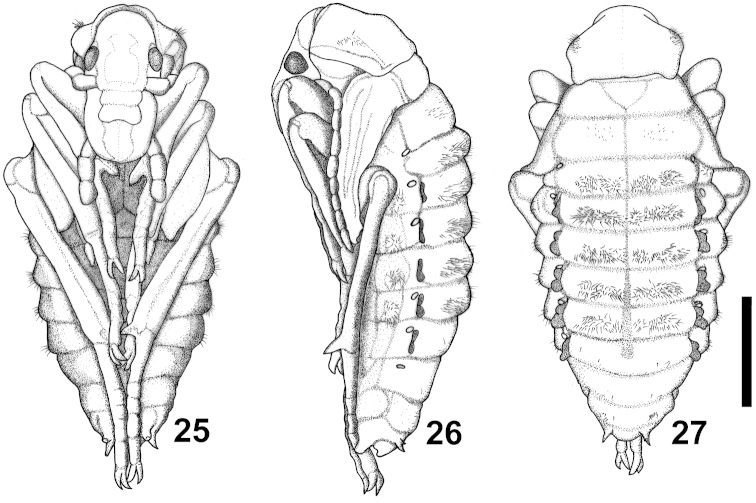
Carabus (Cathoplius) aliai Escalera, 1944 pupa: ventral, lateral and dorsal aspect. Scale bar: 5 mm.

The aspect of the pupa reflected that of the adult. The *Carabus aliai* pupae (Figures 25–27) were smaller (20.0–22.0 × 9.0 mm, length × width) and with a narrower and more elongated thoracic area compared to those of *Carabus stenocephalus ifniensis*, that were larger (22.0–25.0 × 9.5 mm) and with a wider thoracic area. Pupal chaetotaxy was found to be an important character capable of discriminating the two taxa: the *Carabus aliai* pupa showed tufts of scarce and thin setae on the metanotum, tufts of thick, strong and long setae from urotergite I to V, isolated setae on urotergite VI and VII, and two small groups of setae on urotergite VIII. Contrary to this, in the *Carabus stenocephalus ifniensis* pupa, setae on metanotum were extremely scarce, thin and barely visible, while thicker, stronger and longer setae were present from urotergite I to VI and on urotergite VIII but not on urotergite VII (see Busato et al. 2014).

**Figure 28.**
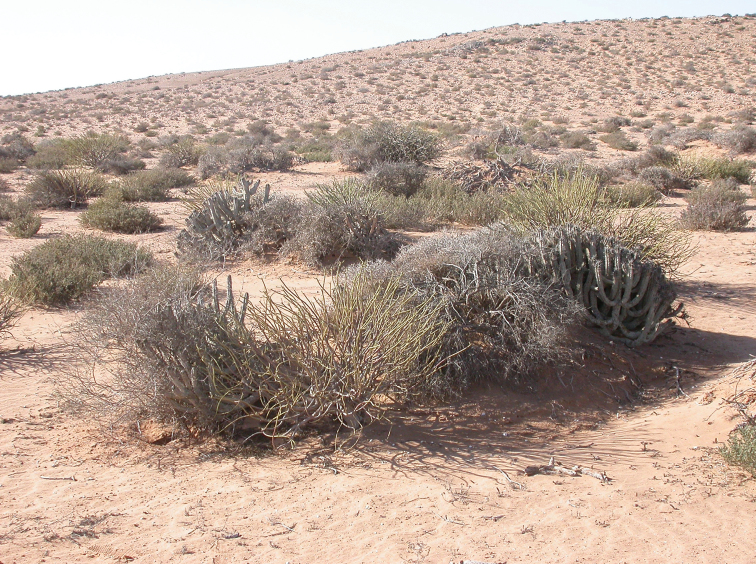
The Carabus (Cathoplius) aliai Escalera, 1944 biotope in Tan-Tan, southern Morocco. Photo by Dr M. Meregalli.

***Field observations*.** The biotope where Carabus (Cathoplius) aliai Escalera, 1944 was found is a sandy and rocky desert area located 15–20 km far from the Atlantic coast, at an elevation comprised between 50 and 200 m a.s.l. (Figure 28). The Tan-Tan northern plateau, in Tan-Tan province, southern Morocco, is a north–south oriented 60 km long and 6–10 km wide mountain (from the Oued Drâa outlet to the Oued Boukhchibia, a tributary of the Oued Chbika). Its xerophilous vegetation is composed mainly by shrubs of *Launaea arborescens* (Battandier, 1888), *Lycium intricatum* Boissier, 1838 and *Tetraena gaetula* (Emberger & Maire, 1928), as well as by the arboreal spurge *Euphorbia regis-jubae* J. Gay, 1847 and the cactus–like spurge *Euphorbia officinarum echinus* (J.D. Hooker & Cosson, 1874). Snails of the family Helicidae inhabiting this vegetation are represented by three species of *Theba* [*Theba subdentata meridionalis* (Sacchi, 1955), *Theba chudeaui* (Germain, 1908) and *Theba sacchii* Gittenberger & Ripken, 1987] and by *Eremina dillwyniana* (Pfeiffer, 1851). *Theba subdentata meridionalis* is the smallest and most common snail, particularly abundant on *Launaea arborescens* and *Euphorbia regis-jubae* shrubs, while *Eremina dillwyniana* is the largest and generally related to *Eremina echinus*. Major carabids cohabiting with *Carabus aliai* are Calosoma (Campalita) maderae (Fabricius, 1775), Calosoma (Caminara) olivieri Dejean, 1831, Scarites (Scallophorites) buparius (Forster, 1771), *Sphodrus leucophthalmus* (Linnaeus, 1758) and Anthia (Termophilum) sexmaculata (Fabricius, 1787), the last three species being more common. The climate in the Tan-Tan area is typical of a steppe–desert, with low rainfall (102 mm/year) exclusively distributed in late autumn and winter (Meregalli 2005). Despite these extreme environmental conditions, the area hosts highly interesting endemic taxa such as *Eurycleonus talamellii* Meregalli, 2005, the largest cleonine weevil of the western Palaearctic region.

At the time of the visit to the Tan-Tan northern plateau in 2010, temperatures ranged from 15–17 °C (night) to 19–25 °C (day). *Carabus aliai* adults and larvae were found from December 17–19 in coincidence with light rains. Adults were seen especially in the late afternoon, coming out from their burrows from underneath the shrubs. Females were preying upon *Theba* snails while staying hidden into the shrubs. Males were seen running quickly from one shrub to another in search of both snails to eat and females to mate. Burrows consisted in oblique holes about 20 cm deep. *Carabus aliai* adults were observed while eating mainly *Theba chudeaui* and secondarily *Theba subdentata meridionalis*, but not *Theba sacchii* and *Eremina dillwyniana*. Larvae were only seen inside *Theba subdentata meridionalis* shells adhering to *Launaea arborescens* branches, at a height from the ground up to 1.5 m. Ecdysis was taking place inside snail shells, where exuviae were normally found. One second instar larva was observed while running on *Launaea arborescens* branches, searching for a new snail to prey upon. Predation did not result in snail falling to the ground, instead the shell remained adherent to the shrub branch during the larval meal.

***Laboratory observations.*** After an adaptation period of one–two hours, during which Carabus (Cathoplius) aliai Escalera, 1944 adults were running frenetically in the terrariums, both males and females started digging their own burrows underneath the moss. Burrows were represented by oblique holes with a diameter of 1.5–2 cm reaching the bottom of the terrarium. Adults were hiding inside the burrows during the day and normally came out for feeding and mating in the late afternoon to early morning. Only when barometric pressure was decreasing, did we detect a prolonged running activity throughout the day, especially in males.

Adults of *Carabus aliai*, fed with *Theba pisana pisana* (O.F. Müller, 1774) snails, were very voracious. Due to their elongate head and narrow pronotum they were able to penetrate deep inside the snail’s shell. Each meal lasted for approximately an hour with males and 2–3 hours with females. Males were killing and partially eating an average of 5–6 snails per night. Female meal totals were 1–3 snails per night. Meals were preferably consumed close to the moss side.

Mating occurred during the night and throughout the whole oviposition period. Males were frequently observed on top of females who were feeding. Each mating lasted for approximately an hour. One day after mating, oviposition took place. Females, after digging an oblique gallery in the soil, started laying eggs by inserting their abdomen at a depth and distance between eggs of 1–1.5 cm. Females layed from 1–6 but up to 12–20 eggs per night. When laying was complete, females sealed the gallery. When laid, eggs were diaphanous white in color and measured 4.5 × 1.5 mm. With time, they gradually became light yellow in color. Their size increased during embryonic development, reaching 5.0 × 2.0 mm. The embryonic development was completed in 15 days. Under laboratory conditions, the duration of the oviposition period in *Carabus aliai* was 100–110 days, from December to March. During this time, the four females laid 277, 282, 298 and 307 eggs, respectively. Two oviposition periods, separated by a short pause (10 days) in January, were noticed. On average, the total productivity of *Carabus aliai* in 95 laying days was 291 eggs/female, with a laying frequency of 3 eggs/night. Following reproduction, all *Carabus aliai* breeders died without undergoing summer diapause.

In contrast to *Carabus aliai*, the reproduction cycle of Carabus (Cathoplius) stenocephalus ifniensis Zarco, 1941 consisted in a first winter and a second spring oviposition cycle, separated from each other by a short burial period (30 days). When the spring cycle ended, breeders buried again and fell into a long summer diapause that lasted until the following winter time, when a scarce number of eggs and viable larvae was produced before breeders died. The average total productivity of *Carabus stenocephalus ifniensis* in 120 laying days was 338 eggs/female (laying frequency of 2.8 eggs/night), subdivided in 242 eggs laid in 75 days during the first oviposition cycle (3.2 eggs/night) and 96 eggs laid in 45 days during the second cycle (2.1 eggs/night).

After hatching, *Carabus aliai* larvae remained in the egg cell for a day to complete sclerification. Once on the surface, newborn larvae begun running around the terrarium searching for snails to feed on. When snails were found, larvae penetrated the shell by keeping their ventral side of the body adhering to the shell walls. Despite their small size [10.0 × 2.0 mm (length × width)], *Carabus aliai* first instar larvae were found to be very aggressive, being able to kill also large *Theba pisana pisana* snails. For the completion of the first instar period, two meals (each lasting 1.5 days) with snails of small size (shell width 10 mm) were necessary. The pre-ecdysis period and ecdysis lasted 2 days. Ecdysis always took place inside snail shells. Exuviae were then found inside the shells.

The *Carabus aliai* second instar larvae (16.0 × 3.5 mm in size) began feeding after integument sclerification, which stood one day. For the completion of the second instar period, three meals (each lasting 1.5 days) with snails of intermediate size (shell width 15 mm) were necessary. The pre-ecdysis period and ecdysis lasted 2.5 days. Ecdysis generally took place inside snail shells or in a pit dug under the shelter or into the soil.

After integument sclerification of one day, the third instar larvae (22.0 × 6.0 mm in size) began feeding again. For the completion of the third instar period, four meals (each lasting 2 days) with snails of large size (shell width 20 mm) were necessary. Then, the mature larvae (28.0 × 6.5 mm in size) stopped feeding and started digging a large cell in the soil for pupating. Inside the pupal cell, pre-pupas (24.0 × 6.5 mm in size) and pupas (21.0 × 9.0 mm in size) occupied 2/3 of the cell length. After emergence from the pupa, *Carabus aliai* neo-adults remained in the pupal cell for about 3 days before rising to the surface.

Under laboratory conditions, the duration of the development cycle from egg fertilization to the rising of neo-adult averaged 2 days shorter in *Carabus aliai* compared to *Carabus stenocephalus ifniensis* (70 vs. 72 days). While embryonic development lasted 15 days in both species, larval development on the surface (from egg hatching to digging of the pupal cell), as well as the burial phase, were one day shorter in *Carabus aliai* than in *Carabus stenocephalus ifniensis* (23 vs. 24 and 32 vs. 33 days, respectively). More specifically, the duration of the first instar period (6 vs. 7 days), the duration of the prepupal period (8 vs. 9 days) and the time between emergence from the pupa and the rising to the surface of neo-adults (3 vs. 5 days) were a little shorter in *Carabus aliai* than in *Carabus stenocephalus ifniensis*, while the duration of the pupal instar was a little longer (21 vs. 19 days) (Table 2).

**Table 2.** Differences between Carabus (Cathoplius) aliai Escalera, 1944 and Carabus (Cathoplius) stenocephalus ifniensis Zarco, 1941 life cycles at the same laboratory conditions [temperature 15/19–17/21 °C (night/day); photoperiod 9–13 hours of daylight].

Life cycle phases	*Carabus aliai* (mean days)	*Carabus stenocephalus ifniensis* (mean days)
Embryonic development:	15	15
- mating – oviposition	1	1
- oviposition – hatching of larvae	14	14
Larval development on the surface:	23	24
- 1^st^ larval instar	6	7
- 2^nd^ larval instar	8	8
- 3^rd^ larval instar	9	9
Burial phase:	32	33
- pre–pupa	8	9
- pupa	21	19
- adult emergence – rising to the surface	3	5
**Duration of development cycle**	**70**	**72**
Completion of adult sclerification	21	21
Time from hardening to burying	2	15
Burial phase (gonad maturation)	–	30
Spring oviposition cycle	–	50 or 0 *
Burial phase (summer diapause)	250	180 or 220 *
Winter oviposition cycle	95	75
End of oviposition – death	15	15
**Life span (larva + pupa + adult)**	**453**	**458**

* Different duration depending on the number of oviposition cycles

Under laboratory conditions, the rising to the surface of *Carabus aliai* neo-adults took place from the beginning of February through mid-April. The mean survival rate from egg to adult was 53% and the final ratio between males and females was of 1:1. Males rose to the surface a little earlier than females. Once on the surface, neo-adults fed on *Theba pisana pisana* snails until their total hardening, which was completed in approximately 3 weeks. During this period, neo-adults were very aggressive, killing a large number of snails throughout the day. Both males and females were partially eating an average of 9–10 snails per day. Even with optimal temperature and humidity conditions, a few days after completing sclerification neo-adults buried themselves and fell into a long summer diapause (8–9 months, starting from April–May) without reproducing. Adults rose to the surface again at the end of November, commencing their oviposition cycle that lasted for 3.5 months until mid-March. Egg production averaged 250 eggs/female. Following reproduction, adults died. Females died a few days after discontinuing oviposition. Males survived approximately 2 weeks longer than females. No feeding attempts were observed during this period. The average life span of *Carabus aliai* under laboratory conditions was 15 months (including 2 months as pre-imaginal stages) (Table 2).

In *Carabus stenocephalus ifniensis* the pattern was found to be similar (the mean survival rate from egg to adult was 52% and the final ratio between males and females was of 1:1), but the reproduction cycle was different. Neo-adults of this taxon, after rising to the surface and completing sclerification in approximately 3 weeks, remained on the surface for about two weeks and then underwent a burial phase that lasted for a month. During this phase, gonad maturation was taking place. By mid-March, precocious neo-adults rose to the surface and started reproducing, but their cycle was short (1.5 months) and egg production was scarce (average of 100 eggs/female). Only one third of neo-adults took part in the spring oviposition cycle, while the majority of individuals remained buried without reproducing. At the end of April–beginning of May, adults that reproduced buried themselves and fell into diapause for 6 months. At mid-November, all individuals rose to the surface and started reproducing. The winter oviposition cycle lasted for 2.5 months and egg production was high (average of 250 eggs/female). Following reproduction, adults died. When considering the two oviposition cycles, the average egg production in *Carabus stenocephalus ifniensis* was 350 eggs/female (100 eggs/female more than in *Carabus aliai*). The average life span of *Carabus stenocephalus ifniensis* under laboratory conditions was 15 months (Table 2).

***Hybrids behavior.*** Hybridization between Carabus (Cathoplius) aliai Escalera, 1944 and Carabus (Cathoplius) stenocephalus ifniensis Zarco, 1941 led to the following results:

**1) *Carabus aliai*** ♂ × ***Carabus stenocephalus ifniensis*** ♀ **crossbreeding (AIF1).** Mating occurred normally, but in a less intensive manner than in pure *Carabus aliai* and *Carabus stenocephalus ifniensis* breeding. Oviposition took place a day after copulation. Females layed from 1 to 4 eggs per night. The average total productivity was 87 eggs/female over a period of 65 days. Following reproduction, adults buried themselves and fell into diapause. Besides a reduced oviposition rate compared to the normal one of the parental taxa, hybridization resulted in a reduced hatching rate and an increased pre-imaginal mortality rate that led to a F1 mean survival rate from egg to adult of 33%, versus 53% in *Carabus aliai* and 52% in *Carabus stenocephalus ifniensis*. The duration of the development cycle in hybrid AIF1 was 74 days, subdivided into 15 days for embryonic development, 25 days for larval development on the surface and 34 days for the burial phase. Sclerification of neo-adults was completed in about 3 weeks. Afterwards, neo-adults remained on the surface for 3–4 weeks before falling into diapause. Hybrids had a sturdiness similar to that of pure *Carabus aliai* or *Carabus stenocephalus ifniensis* specimens, with a life span of 12–15 months.

The F1 crossbreeding (AIF1 × AIF1) led to a reduced fecundity rate (41 eggs/female over a period of 45 days), to neonatal mortality with scarcely viable F2 pre-imaginal stages (70% mortality rate for first instar larvae, 48% for second instar larvae, 32% for third instar larvae, 80% for pre-pupas and pupas) and to stillbirth with non-viable F2 imagoes (100% mortality rate inside the pupal cell, without any rising to the surface of neo-adults).

**2) *Carabus stenocephalus ifniensis*** ♂ × ***Carabus aliai*** ♀ **crossbreeding (AIF2).** The behavior was found to be similar to that seen in the previous combination. The average total productivity was 69 eggs/female over a period of 75 days. Two oviposition periods, separated by a short pause (15 days), were noticed. Also in this case, hybridization was responsible for a reduced hatching rate and an increased pre-imaginal mortality rate that led to a F1 mean survival rate from egg to adult of 31%. The duration of the development cycle in hybrid AIF2 was 72 days, subdivided into 15 days for embryonic development, 24 days for larval development on the surface and 33 days for the burial phase. After sclerification, neo-adults remained on the surface for about 2 weeks before falling into diapause.

The F1 crossbreeding (AIF2 × AIF2) led to a reduced fecundity rate (33 eggs/female over a period of 53 days), to neonatal mortality with scarcely viable F2 pre-imaginal stages (82% mortality rate for first instar larvae, 50% for second instar larvae, 34% for third instar larvae, 84% for pre-pupas and pupas) and to stillbirth with non-viable F2 imagoes (100% mortality rate inside the pupal cell).

## Discussion

***Morphology.*** The subgenus *Cathoplius* within the Genus *Carabus* includes ground beetles characterized by strongly sclerified integument, black in color, brachypterous, with elytra that are joined along the suture, an achetous, ellipsoidal, silphoid or cychrized pronotum, and a narrow and very elongate head (Busato et al. 2014). According to their close geographic vicariance and their clinal morphological variation along the Atlantic Moroccan coast (from stocky, tenebrionid-like northern forms to more elongate, cychrized southern forms), *Cathoplius* are considered as belonging to a single species [*Carabus asperatus* (Dejean, 1826)] in some catalogues (Březina 1994; 1999; Bousquet et al. 2003; Lorenz 2005). However, in most taxonomic treatments, *Cathoplius* taxa are ascribed to two species: *Carabus asperatus*, monotypic, with a northern distribution, and *Carabus stenocephalus* Lucas, 1866, polytypic, with a southern distribution, *Carabus aliai* Escalera, 1944 being considered as the southernmost subspecies of the latter (Antoine 1955; Kocher 1963; Deuve 1994; 2004; 2012; 2014). The *Carabus stenocephalus* taxonomic subdivision into subspecies was adopted after examining a scarce number of individuals coming from the southern parts of the distributional area (Zarco 1941; Escalera 1944; Español 1944; Mateu 1947). This fact has led to the statement of considering *Carabus aliai* as a simple subspecies of *Carabus stenocephalus* and not as a separate species.

The morphological differences between *Carabus aliai* and *Carabus stenocephalus* (*sensu lato*) are remarkable. The pronotum profile and the elytral sculpture are very peculiar. Despite their close range of distribution (only 50 km are dividing Sidi Ifni Beach from Plage Blanche), differences are striking when *Carabus aliai* adults are compared to those of *Carabus stenocephalus ifniensis* Zarco, 1941. These differences include a smaller size, a much slender silhouette, a more brilliant aspect, longer legs, and a wider and a little more curved apex of the median lobe of the aedeagus. Differences are also present referring to pre-imaginal stages: *Carabus aliai* larvae are much smaller than those of *Carabus stenocephalus ifniensis* (9.5 × 2.0 mm vs. 11.0 × 2.5 mm in newborn larvae) and the *Carabus aliai* pupa is also smaller, with a narrower thoracic area and a different chaetotaxy. Both imaginal and pre-imaginal features are an index of the specific differentiation between the two taxa.

***Life cycle.*** The 2010 raining season in the Tan-Tan area began on November 30, with a storm (17 mm of rain at a temperature of 16–23 °C) that induced the rising of Carabus (Cathoplius) aliai Escalera, 1944 adults from their summer diapause. Oviposition occurred some days later, with eggs hatching on December 13–14 and first ecdysis on December 18–19, when second instar larvae were found. The second ecdysis occurred at the laboratory on December 28–31 and the burying of third instar larvae on January 7–9, with rising to the surface of neo-adults on February 8–15. Out of the ten wild larvae found, two died as second instar and two as third instar, and out of the six surviving adults, four were males and two were females. The 60% survival rate noticed for wild larvae matches the mean survival rate of 53% obtained at the laboratory from egg to adult, but the ratio between males and females does not (2:1 in nature vs. 1:1 at the laboratory). This is probably due to the fact that, after emergence, precocious males are rising to the surface earlier than first females.

In mid-March *Carabus aliai* neo-adults buried themselves and fell into a summer diapause that lasted until late autumn, when terrariums were abundantly watered. When watering was carried out during late spring or in summer, adults were rising to the surface, feeding for some days and burying again without mating. The beginning of reproduction in representatives of subgenus *Cathoplius* depends exclusively on the start of the natural raining season (Busato et al. 2014). In *Carabus stenocephalus ifniensis* Zarco, 1941 the raining season normally starts at the beginning of November, while in *Carabus aliai* it starts at the beginning of December. The reason for this difference is that on the western slopes of the Anti-Atlas chain, where Sidi Ifni is located and where *Carabus stenocephalus ifniensis* dwells, the first rain usually falls a month earlier than in the desert area around Tan-Tan.

While under laboratory conditions the oviposition period in *Carabus aliai* lasted for over three months, in nature this is not the normal case. Aridity and cold weather at night are responsible for a lack of active *Theba* snails, and correspondingly no activity of *Carabus aliai* adults and larvae is present in January in the Tan-Tan area. In nature, the oviposition period probably lasts for a month, from the beginning to the end of December, and after reproduction all adults die. The *Carabus aliai* life cycle can therefore be placed into the winter breeders with short larval development type *sensu* Paarmann (1979) and into the annual univoltine winter semelparous type *sensu* Matalin (2007). The last breeding type is well adapted to desert conditions with short winter rains. Contrary to this, the *Carabus stenocephalus ifniensis* life cycle belongs to the late autumn breeders type and is iteroparous, where adults who bred in late autumn become active again and breed a second time in early spring before summer diapause.

One of the most striking features in representatives of the subgenus *Cathoplius* is their high fecundity rate. Species belonging to this subgenus are one of the most prolific among the known ground beetles (Busato et al. 2014). In our experiments, *Carabus aliai* females laid 250–300 eggs in 100 days that resulted in 125–150 neo-adults. In nature, the number of laid eggs and the consequent number of neo–adults is certainly lower, due to the shortened oviposition period and to predation. Ordinarily, each female should lay an average of 80 eggs in a month resulting in 40 neo-adults.

The mean duration of the development cycle, from egg fertilization to the rising of neo-adults, was found to be shorter in *Carabus aliai* than in *Carabus stenocephalus ifniensis*. Results of the present study are in agreement with those obtained by Busato et al. (2014) where, at a constant temperature of 21±1 °C, the development cycle was a day shorter in *Carabus aliai* than in *Carabus stenocephalus ifniensis* (69 and 70 days, respectively). At temperatures varying from 15/19 to 17/21 °C (night/day) the development cycle was a little longer in both taxa, but indeed shorter in *Carabus aliai* than in *Carabus stenocephalus ifniensis* (70 vs. 72 days). In nature, this fact is likely due to the desert habitat of the Tan-Tan area, where snails activity is reduced to shorter periods compared to that on the Sidi Ifni beach.

From the behavioral point of view, *Carabus aliai* is a typical sabulicolous steppe wandering species (Mateu 1947). A remarkable and intensive running activity, more evident in males, was noticed both in the field and in terrariums. Moreover, adults dig burrows underneath shrubs where they hide during the day for protection from dehydration and predation. This way of life is common to other cohabiting steppe wandering species, such as Calosoma (Campalita) maderae (Fabricius, 1775) and Calosoma (Caminara) olivieri Dejean, 1831 (Ghittino, personal observations). Contrary to *Carabus aliai*, running activity in *Carabus stenocephalus ifniensis* was found infrequently. *Carabus stenocephalus ifniensis* is a more sedentary taxon, carrying out the major part of its life inside the *Lycium intricatum* and *Tetraena gaetula* shrubs that are present on the Sidi Ifni coastal area, where snails belonging to the species *Theba subdentata meridionalis* (Sacchi, 1955) and *Theba solimae* (Sacchi, 1955) are dwelling. This sedentary behavior was confirmed at a laboratory level, where running activity in terrariums was rarely observed even when barometric pressure was decreasing.

In the field, *Carabus aliai* adults were observed mainly feeding upon *Theba chudeaui* (Germain, 1908) and secondarily upon *Theba subdentata meridionalis* snails. This is likely due to the major shell width, the larger aperture and the lack of a parietal denticle in *Theba chudeaui*. The fact that *Eremina dillwyniana* (Pfeiffer, 1851) snails, which are abundant in the Tan-Tan area, were not eaten by *Carabus aliai* is an additional proof that representatives of subgenus *Cathoplius* feed exclusively on *Theba* snails. First and second instar *Carabus aliai* larvae were only found inside *Theba subdentata meridionalis* shells adhering to *Launaea arborescens* branches. The completion of the first pre-imaginal stage on vegetation is probably essential for *Carabus aliai* larval survival, in order to avoid predation from associated carabids. At the Tan-Tan biotope several very aggressive species are present, such as Anthia (Termophilum) sexmaculata (Fabricius, 1787), Scarites (Scallophorites) buparius (Forster, 1771) and *Sphodrus leucophthalmus* (Linnaeus, 1758) (Ghittino, personal observations). *Theba subdentata meridionalis* snails are probably the prey of choice for *Carabus aliai* larvae for their minor shell width, the smaller aperture and the presence of a well developed parietal denticle that better protects larvae from aggressions.

As predators of live *Theba* snails, *Cathoplius* should play an important role in the ecosystem by reducing *Theba* proliferation which can be detrimental to both conservation of the scarce vegetation in arid areas and to animal husbandry, by preventing important livestock parasitic diseases among grazing land animals (Busato et al. 2014). *Theba* snails are involved in the life cycle of sheep and goat lungworms by harboring the infective stage of some protostrongylid nematodes (Cabaret 1987; Grewal et al. 2003). Sheep and goat breeding is a common activity in the Moroccan Saharan provinces. Parasitic diseases affecting sheep and goat lungs are frequent in this area and can be diagnosed both clinically and at slaughtering (Ghittino, personal observations).

We could not find any reports of *Theba subdentata meridionalis* or *Theba chudeaui* acting as intermediate hosts for sheep and goat lungworms but the presence of the pathology in animals and the observation of worm stages in snails are evidence of their involvement in the disease life cycle. Differently from other local snails, *Theba subdentata meridionalis* and *Theba chudeaui* live on shrubs that are particularly appreciated by sheep and goats (e.g. *Launaea arborescens* and *Lycium intricatum*) which then can be easily infected through accidental ingestion of parasitized snails.

Under laboratory conditions, *Carabus aliai* adults and larvae were found to be very aggressive against *Theba* snails. Adults killed up to 5–6 snails per night over a period of three months. For completing the development cycle, each larva fed on an average of nine snails and to reach complete sclerification neo-adults killed up to 9–10 snails per day over a period of three weeks. These data indicate that *Carabus aliai* is an efficacious natural enemy of *Theba* snails that can also reduce the impact of some economically important ruminant diseases. Conservation of *Carabus aliai* in southern Morocco is therefore recommended.

***Hybridization.*** The results of our hybridization experiments between Carabus (Cathoplius) aliai Escalera, 1944 and Carabus (Cathoplius) stenocephalus ifniensis Zarco, 1941 are in agreement with those obtained by Busato et al. (2014). The few discrepancies in the current study are likely due to the different breeding temperatures applied. Despite a normal mating attitude of parents, a shorter oviposition period and a reduced number of laid eggs was noticed in both crossbreeding combinations compared to what observed in pure *Carabus aliai* and *Carabus stenocephalus ifniensis* breeding. This fact was more evident for the AIF2 (*Carabus stenocephalus ifniensis* ♂ × *Carabus aliai* ♀) than for the AIF1 (*Carabus aliai* ♂ × *Carabus stenocephalus ifniensis* ♀) combination. Besides this, F1 hybrids underwent a high mortality rate during embryonic, larval and pupal development that led to a 40% reduction of the final survival rate compared to that of *Carabus aliai*. F1 hybrids showed also a longer duration of their development cycle (72 days in AIF2 hybrid and 74 days in AIF1 hybrid vs. 70 days in *Carabus aliai*).

The F1 hybrids crossbreeding (AIF1♂ × AIF1♀ and AIF2♂ × AIF2♀) led to a further reduction of laid eggs and a very high mortality rate during embryonic, larval and pupal development. This was also more evident in the AIF2 than in the AIF1 combination. Only a few F2 immature imagoes were obtained (3 out of 120 larvae for AIF1 and 1 out of 100 larvae for AIF2), but these specimens died in the pupal cell within 10 days from emergence. Since no vital neo-adults were available, it was impossible to carry out subsequent F2 hybrids crossbreeding.

Through our crossbreeding experiments, that employed a high number of F1 hybrids (4 mating pairs for each combination) maintained at natural conditions with regards to temperature and photoperiod, we demonstrated that a marked reproductive isolation between *Carabus aliai* and *Carabus stenocephalus* is present. This isolation corroborates the distinction at a species level of the two taxa and is similar to the isolation observed between *Carabus stenocephalus* and *Carabus asperatus* (Busato et al. 2014).

## Conclusions

Data generated in this study, together with those obtained by Busato et al. (2014), clearly indicate that Carabus (Cathoplius) aliai Escalera, 1944 is an independent species and not a mere subspecies of Carabus (Cathoplius) stenocephalus Lucas, 1866. Despite their close geographic distributional area, and the fact that they are geographical substitutes, the morphology and biology are very different between *Carabus aliai* and *Carabus stenocephalus ifniensis* Zarco, 1941. In *Carabus aliai*, adults are characterized by a shorter size and a more slender silhouette, larvae are smaller and pupae have a different chaetotaxy. The life cycle in *Carabus aliai* belongs to the annual univoltine winter semelparous type, where adults of the parental generation die after the breeding period ends. The duration of the development cycle, from eggs fertilization to the rising of neo-adults, is shorter in *Carabus aliai* then in *Carabus stenocephalus ifniensis* [70 vs. 72 days at temperatures varying from 15/19 to 17/21 °C (night/day)]. As a way of life, *Carabus aliai* is a typical sabulicolous steppe wandering species with an intensive running activity, while *Carabus stenocephalus ifniensis* is a more sedentary taxon carrying out the major part of its life inside shrubs. Feeding on *Theba* snails is common to both species and centers mainly on the predation of *Theba subdentata meridionalis* (Sacchi, 1955) and *Theba solimae* (Sacchi, 1955) (in the case of *Carabus stenocephalus ifniensis*) and on *Theba subdentata meridionalis* and *Theba chudeaui* (Germain, 1908) (in the case of *Carabus aliai*). Besides the different morphology and biology, results of crossbreeding experiments are indicative of a marked reproductive isolation between *Carabus aliai* and *Carabus stenocephalus*. When F1 hybrids are crossed with one another, progenies undergo a very high mortality during embryonic, larval and pupal development. Only a few F2 hybrids can be obtained, but these specimens usually die within some days from emergence.

**Figure 29.**
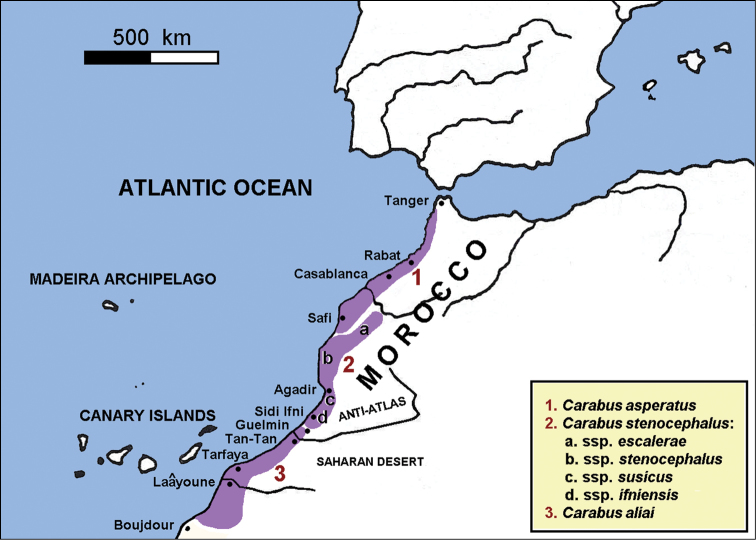
Distribution map of Carabus (Cathoplius) aliai Escalera, 1944 and its sister species.

According to these results, three *Cathoplius* species are spread along the Moroccan Atlantic coast: Carabus (Cathoplius) asperatus (Dejean, 1826) in the north, *Carabus stenocephalus* Lucas, 1866 in the center and *Carabus aliai* Escalera, 1944 in the south (Figure 29). *Carabus aliai* is distributed in an area south of the Anti-Atlas chain, from Plage Blanche (Guelmim province) to Lemsid and Bou Kra (south of Laâyoune/north of Boujdour). This area is approximately 450 km in length. *Carabus aliai* is therefore both a Saharan desert endemic and an Atlantic species. Moreover, it is the southernmost *Carabus* species of the western Palaearctic region.

A distributional gap, probably due to the lack of *ad hoc* investigations, is actually present between northern and southern *Carabus aliai* populations. Northern populations dwell in Guelmim (Plage Blanche) and Tan-Tan (Ben Khlil, Tan-Tan, Abteh) provinces, while southern populations are spread from present Laâyoune (Izik plateau, Sabkhat Tislatine, Bou Kra) to Boujdour (Lemsid, Metmarfag) provinces. The 200 km area in between, corresponding to present Tarfaya province, is represented by a very sandy desert with a few spots suitable for *Carabus aliai* such those around Akhfennir, Daoura and El Hagounia. Searches of this area during the favorable season will probably allow the identification of new *Carabus aliai* populations.
